# Lipidomic Profiling of Lung Pleural Effusion Identifies Unique Metabotype for EGFR Mutants in Non-Small Cell Lung Cancer

**DOI:** 10.1038/srep35110

**Published:** 2016-10-14

**Authors:** Ying Swan Ho, Lian Yee Yip, Nurhidayah Basri, Vivian Su Hui Chong, Chin Chye Teo, Eddy Tan, Kah Ling Lim, Gek San Tan, Xulei Yang, Si Yong Yeo, Mariko Si Yue Koh, Anantham Devanand, Angela Takano, Eng Huat Tan, Daniel Shao Weng Tan, Tony Kiat Hon Lim

**Affiliations:** 1 Bioprocessing Technology Institute Agency for Science, Technology and Research (A*STAR), 20 Biopolis Way, #06-01 Centros, Singapore 138668, Singapore; 2 Department of Pathology Singapore General Hospital 20 College Road, Academia, Level 10, Singapore 169856, Singapore; 3 Institute of High Performance Computing, Agency for Science, Technology and Research (A*STAR), 1 Fusionopolis way, #16-16 Connexis, Singapore 138632, Singapore; 4 Department of Respiratory and Critical Care Medicine, Singapore General Hospital, Outram Rd, Singapore 169608; 5 National Cancer Centre, 11 Hospital Drive, Singapore 169610, Singapore; 6 Genome Institute of Singapore, Agency for Science, Technology and Research (A*STAR), 20 Biopolis Way, #06-01 Centros, Singapore 138668, Singapore

## Abstract

Cytology and histology forms the cornerstone for the diagnosis of non-small cell lung cancer (NSCLC) but obtaining sufficient tumour cells or tissue biopsies for these tests remains a challenge. We investigate the lipidome of lung pleural effusion (PE) for unique metabolic signatures to discriminate benign versus malignant PE and EGFR versus non-EGFR malignant subgroups to identify novel diagnostic markers that is independent of tumour cell availability. Using liquid chromatography mass spectrometry, we profiled the lipidomes of the PE of 30 benign and 41 malignant cases with or without EGFR mutation. Unsupervised principal component analysis revealed distinctive differences between the lipidomes of benign and malignant PE as well as between EGFR mutants and non-EGFR mutants. Docosapentaenoic acid and Docosahexaenoic acid gave superior sensitivity and specificity for detecting NSCLC when used singly. Additionally, several 20- and 22- carbon polyunsaturated fatty acids and phospholipid species were significantly elevated in the EGFR mutants compared to non-EGFR mutants. A 7-lipid panel showed great promise in the stratification of EGFR from non-EGFR malignant PE. Our data revealed novel lipid candidate markers in the non-cellular fraction of PE that holds potential to aid the diagnosis of benign, EGFR mutation positive and negative NSCLC.

Lung cancer is the leading cause of cancer-related mortality worldwide, with non-small cell lung cancer (NSCLC) being the predominant form of the disease, accounting for ~85–90% of all cases^1^,^2^. Currently, small biopsy and cytological examination of malignant cells forms a cornerstone in the diagnosis of lung cancer^3^. Beyond establishing a definitive diagnosis of NSCLC, the American College of Chest Physicians recommends such diagnostic workup to classify the subtypes of lung cancer (e.g. adenocarcinoma versus squamous) and the molecular status based on specific gene mutations as that would facilitate the prescription of personalized therapy for individual patients by clinicians^3^,^4^. In particular, specific mutations in the epidermal growth factor receptor (EGFR) have been reported to be one of the top driver oncogenes in NSCLC, with a prevalence of 9–23%^5^. NSCLC patients harbouring EGFR mutations are known to be more responsive to tyrosine kinase inhibitors (TKIs) such as gefitinib and erlotinib, making such medications the first-line therapy instead of standard chemotherapy^6^.

Adequate tumour cell and tissue acquisition is paramount for diagnosis, histologic and molecular characterization of NSCLC. However, the limited availability of cells and tissues for these diagnostic workups is an on-going challenge for pathologists. The presence of pleural effusion (PE) is commonly observed in the initial diagnosis of lung cancer with an occurrence of 7 to 30% of all lung cancer cases^7^. Cytology of malignant cells from PE facilitates diagnosis of NSCLC. Lung PE, however, can also be a manifestation of benign inflammatory conditions including pneumonia, tuberculosis and pulmonary disorders. While cytological examination of PE aids in diagnosis of NSCLC, it is noteworthy that the diagnostic performances of cytology is dependent on the tumour type, tumour burden in the pleural space and the cytologist’s expertise^8^. In PE samples with low cell yields, diagnostic accuracy can be compromised, resulting in false-negative rates of more than 30%^9^,^10^. Consequently, the identification of molecular biomarkers independent of tumour cell and tissue is pertinent to complement and circumvent the challenge in the diagnosis of NSCLC.

To date, several efforts to identify alternative strategies using PE supernatant to complement the diagnostic workup for NSCLC have been reported. Several of these studies examine the diagnostic potential of protein-based molecules (e.g. carcinoembryonic antigen (CEA) and α-fetoprotein) and carbohydrate antigens (e.g. CA125 and CA 9-9), which are commonly present in other cancer types^11^,^12^,^13^. However, the outcomes of using abovementioned protein molecules as candidate malignancy markers are mixed as studies revealed large variations in their sensitivities for diagnosis. At present, molecular characterization of EGFR mutations is performed using DNA extracts from tumour tissue specimens. While DNA extracted from malignant PE supernatant has been suggested as an alternative, the large variation in the quantity and quality of the DNA present in such samples can compromise the sensitivity of this approach. As observed in a recent study performed by Liu and co-workers, the malignant PE supernatant was reported to have a low sensitivity of 63.6% in comparison to the tumour tissue^14^. Henceforth, the authors recommended that DNA extracted from malignant PE supernatant should not be used for mutation testing if tumour tissue is available. Nevertheless, it is noteworthy that other studies reported favourable clinical usefulness of cell-free DNA from blood for the detection of EGFR mutations as supported by promising diagnostic performance (sensitivity up to ~80% and specificity of 100%)^15^,^16^. Collectively, these studies demonstrated some of the efforts to identify surrogate markers in liquid biopsy that are indicative of the mutation subtype so as to overcome the issue of limited availability of tumour tissue for genotyping.

More recently, metabolic reprogramming involving deregulated cellular energetics of tumour is enshrined as one of the hallmarks of cancer^17^,^18^. In the context of NSCLC, metabolic profiling using NMR detected elevated levels of branched chain amino acids, lactate and alanine and suppressed levels of glucose, trimethylamine-N-oxide and creatinine in malignant PE^19^. Using an untargeted LC-MS approach, Lam *et al*. observed significant elevation of fatty acids FA(16:0), FA(18:0), FA(18:1) and (FA18:2), as well as a decrease in a ceramide species Cer(d18:1/16:0) in malignant PE^20^. Oleic acid (FA(18:1)) was found to be the best individual differentiator between benign tuberculosis PE samples and malignant lung adenocarcinoma PE, with an area-under-curve (AUC) value of 0.962 from receiver operating characteristic (ROC) analysis.

While these metabolic studies illuminated the potential of using PE-derived metabolites as markers for malignancy in NSCLC, the alterations in the metabolic phenotype (metabotype) in PE and their utility in the diagnosis of NSCLC is not well studied. The identification of fatty acid marker metabolites in the study by Lam *et al*. coincide with the lipid reprogramming phenomenon in cancer biology that is gaining increasing recognition in recent years^20^,^21^. Collectively, this underscored the value in scrutinizing the lipidome of PE in NSCLC diagnosis. It is noteworthy that while interesting lipid candidate markers were identified in the Lam *et al*. study, their extraction method (using 1:1:1 v/v/v of acetone/ethanol/methanol) is not well optimized to interrogate the PE lipidome. Additionally, there has been no other follow up studies to evaluate the validity of the key findings in additional patient cohorts. Therefore, the primary objective of this study is to compare the lipidomes of the non-cellular fraction of lung PE derived from benign and malignant patients. To our knowledge, there is no study to date that investigates the utility of small molecules in biofluid as potential surrogate markers for the stratification of mutational subtypes in lung cancer. Hence, our secondary objective aims to identify lipid species with the ability to distinguish malignant PE from NSCLC patients with and without EGFR mutations.

## Results

### Pleural effusion lipidomes

We undertook an in-depth LC-MS based lipidomic analysis of 71 PE samples (30 benign, 41 malignant–19 EGFR mutant, 17 non-EGFR mutant and 5 with unknown EGFR status) (Table 1). The enrolled subjects comprising of 61 Chinese, 5 Malay, 4 Indian and 1 other ethnicity, generally reflecting the predominant distribution of the major ethnic group in Singapore. No statistically significant differences were identified in terms of age (p-value = 0.09), gender (p-value = 0.15), smoking status (p-value = 0.46) and ethnicity (p-value = 0.25) between the benign and malignant patients.

From the lipidomic analysis of benign and malignant PE samples, we detected a diverse range of lipid species in the human PE (Table S1, Figure S1). These species were identified based on authentic standards or by comparing the characteristic MS^2^ spectra of the respective lipid classes with online mass spectral databases^22^. The list of identified lipids includes long chain fatty acids, sphingolipids, phospholipids and triacylglycerols.

### Lipidomic analysis highlighted key differences in benign and malignant PE

**Figure 1:**
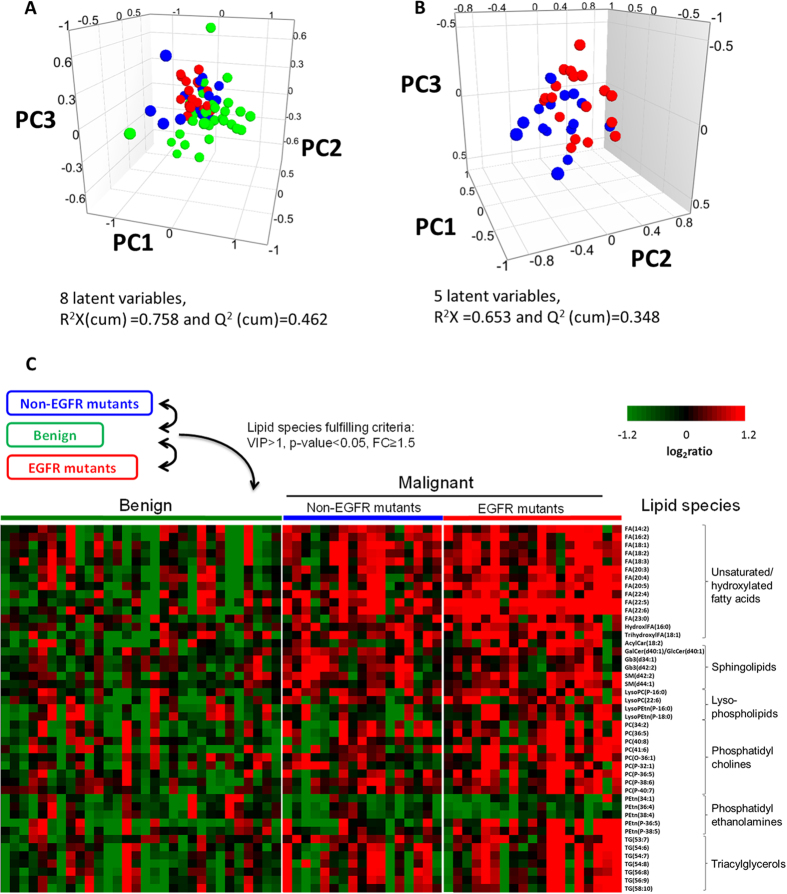
Principal component analysis scores plots for (**A**) benign (n = 30) and malignant lung PE (n = 36), and the PE of (**B**) EGFR mutant (n = 19) and non-EGFR mutant (n = 17) collected from the malignant patients. Green, blue and red circles represent the benign, malignant non-EGFR mutant and malignant EGFR mutant PE respectively. (**C**) Heat map of differential lipid metabolites derived from individual pairwise comparisons between benign, non-EGFR mutant and EGFR mutant PE samples. All represented species are statistically significant (VIP > 1, p-value < 0.05, fold change (FC) ≥ 1.5) for at least one of the pairwise comparisons. Metabolites are grouped according to their lipid classes.

To compare the composition of the lipidomes between the benign and malignant patients, we performed PCA analysis to identify any intrinsic clustering pattern of the PE samples. The PCA analysis of the PE lipidomes revealed distinctive clustering of the benign and malignant cases, indicating the existence of metabolic differences between these two groups (Fig. 1A). Interestingly, closer scrutiny of the PCA scores plot for the malignant PE revealed further compositional differences in the lipidomes between the EGFR mutant and non-EGFR mutant groups within the malignant class (Fig. 1B).

In view of the heterogeneity in the malignant lipidomes between non-EGFR and EGFR mutants, we performed two separate pairwise supervised multivariate analyses using OPLS-DA subsequently to build models to identify potential lipid differentiators with VIP > 1 between: (i) benign vs EGFR mutant and (ii) benign vs non-EGFR mutant cases. Additionally, each pairwise multivariate analysis was supplemented by the use of univariate statistical tools (Mann-Whitney U test, fold change analysis). From these analysis, we identified 45 lipid species satisfying the following criteria: VIP > 1^23^, p-value < 0.05 and an average ratio ≥1.5, in at least one of the pair-wise comparison analysis (Fig. 1C).

Unsaturated fatty acids, phospholipids and sphingolipids constituted some of the major lipid classes discriminating between the malignant from benign PE samples (Figs 1C, and S2). Within the benign patients, we observed no clear differentiation in the abundance of these lipid markers between the benign infective (pneumonia and tuberculosis) and benign non-infective (cardiopulmonary congestion) PE samples for markers indicated in the heat map. These lipid species, however, were significantly elevated in the malignant PE of NSCLC patients compared with benign PE. Consistent with the earlier observation of a heterogeneous malignant lipidomic profile, the heat map analysis further illustrated the metabolic differences present in malignant lung PE associated with their genotype (presence/absence of EGFR mutation) (Fig. 1C). For instance, ether-linked phospholipids such as PC(P-36:5) and PEtn(P-38:5) were found to be statistically different between benign and EGFR mutant PE samples but not between benign and non-EGFR mutant cases. Conversely, glycosylated ceramide species including Gb3(42:2) and Gb3(34:1) were found to be significantly elevated in non-EGFR mutant cases, but not in PE cases with EGFR mutation.

**Figure 2:**
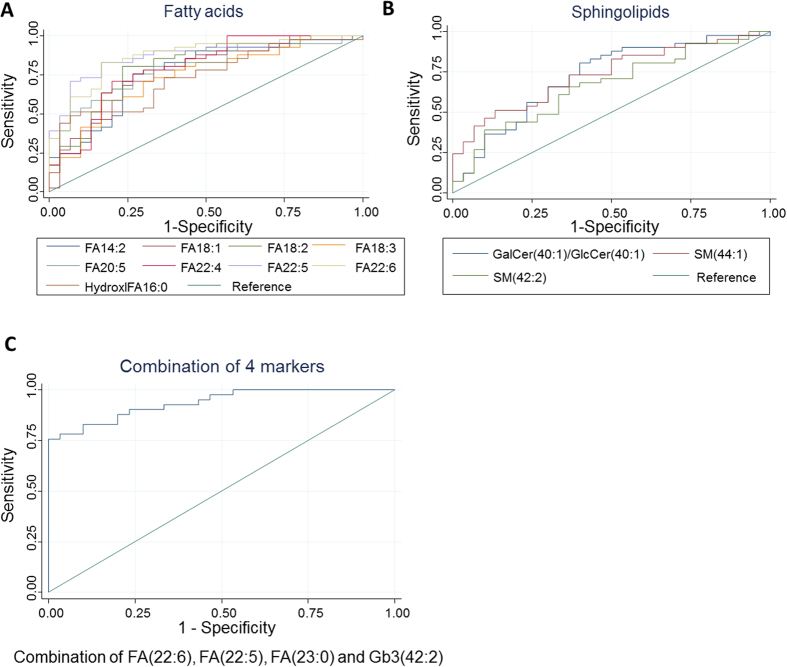
Diagnostic performance of lipid markers in discriminating pleural effusion from malignant NSCLC patients (n = 41) from benign subjects (n = 30). ROC curves of malignant versus benign subjects for individual PE lipid markers in the class of (**A**) fatty acids (**B**) sphingolipids. (**C**) ROC curves of malignant versus benign subjects for an optimal combination of 4 lipid malignancy markers from SVM modelling.

To account for the unique metabotype dependent on the driver mutation for NSCLC, we screened for only lipid species satisfying both the abovementioned criteria for “benign vs EGFR mutant” and “benign vs non-EGFR mutants” comparisons as candidate malignancy markers (Table 2). These identified candidates include a large number of unsaturated fatty acids, in addition to specific ceramide and sphingomyelin species. Subsequently, we assessed the diagnostic performance of this panel of 12 malignancy lipid species using ROC curve analysis (Fig. 2A,B, Table 2). Each of these lipid malignancy markers was able to discriminate between the malignant and benign groups with AUC values ranging from 0.66–0.87, sensitivity (SN) of 63.3–82.9%, specificity (SP) of 60.0–83.3% and accuracy (ACC) of 64.8–83.1%. Individual ROC analysis performed on each candidate indicated that the polyunsaturated fatty acids (PUFAs) (e.g. FA(22:5), FA(22:6)) gave the best performance as malignancy markers and should be further evaluated in larger scale studies. Combining an optimal number of four malignancy markers comprising of FA(22:6), FA(22:5), FA(23:0) and Gb3(42:2) derived from SVM modelling into a single panel gave a comparable performance than when using the biomarkers alone (AUC = 0.94; SN = 82.9%; SP = 90.0%; ACC = 85.9%) (Fig. 2C).

### EGFR mutants exhibited greater metabolic derangement compared to non-EGFR mutants

**Figure 3:**
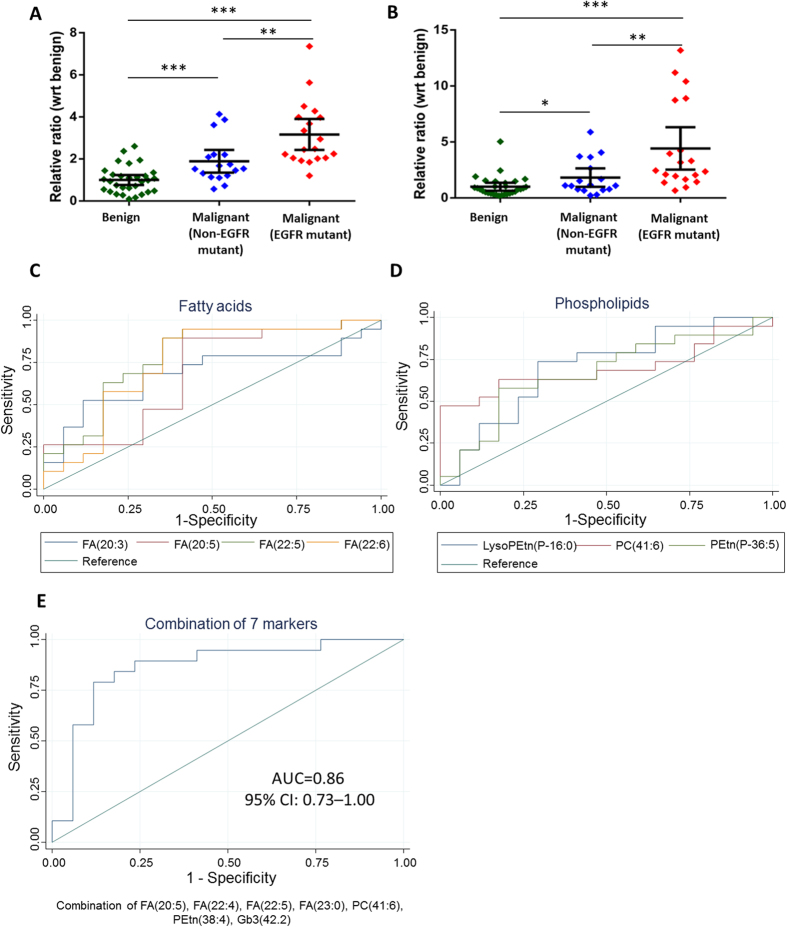
Dot plots describing the relative levels of (**A**) FA(22:6), (**B**) FA(20:5) in benign (green), non-EGFR mutant (blue) and EGFR mutant (red) PE samples. p-value calculated based on Mann-Whitney U test, where *denotes p < 0.05, **denotes p < 0.01, ***denotes p < 0.001. ROC curves of non-EGFR mutant versus EGFR mutant subjects for individual PE lipid markers in the class of (**C**) fatty acids and (**D**) phospholipids. (**E**) ROC curves of non-EGFR mutant versus EGFR mutant subjects for an optimal combination of 7 lipid malignancy markers from SVM modelling.

From the earlier analyses, we noticed that the candidate malignancy markers were predominantly in higher abundance in the PE of the EGFR mutants compared to the non-EGFR mutants (Fig. 1C). The most prominent observations were the increase in levels of PUFAs comprising of 20 and 22 carbons, as well as ether-linked phosphatidylethanolamine (PEtn) and its corresponding lysoPEtn species in EGFR mutants. These trends are exemplified in Fig. 3A,B for FA(22:6) and FA(20:5), illustrating that the EGFR mutants exhibit greater derangement in lipid metabolism compared to non-EGFR mutants. To further understand the influence of NSCLC driver mutations (EGFR/non-EGFR) on the lipidomic profile, we performed a combination of Mann-Whitney U test, OPLS-DA analysis and ROC analyses to identify lipid species that discriminated the EGFR mutants from the non-EGFR mutants (Table 3). Interestingly, we observed that PUFAs comprising of 20 and 22 carbons and PEtn species were significantly elevated in EGFR mutants by 1.6 to 2.6-fold compared to non-EGFR mutant cases (Table 3). Each of these lipid species was able to discriminate between the EGFR mutants and non-EGFR mutants with AUC ranging from 0.67–0.78, SN of 63.2–89.5%, SP of 58.8–82.4% and ACC of 66.7–77.8% (Table 3, Figs 3C,D). A combination of an optimal number of 7 lipid species comprising of FA(20:5), FA(22:4), FA(22:5), FA(23:0), PC(41:6), PEtn(38:4), Gb3(42.2) based on SVM modelling, resulted in a more superior performance compared to using the biomarkers alone (AUC = 0.86; SN = 84.2%; SP = 82.4%; ACC = 83.3%) (Fig. 3E).

## Discussion

The motivation for this study stemmed from challenges in the diagnosis of NSCLC, which is highly dependent on the availability of tissue biopsy or cells for the standard testing of malignancy and mutation status (e.g. EGFR, ALK mutations). The limited tumour tissue and cell availability, the low and variable sensitivity of cytology (ranging from 43–91%) and the significant false-negative rate in the event of insufficient cell numbers from PE provided the impetus to investigate novel, complementary diagnostic markers^4^,^10^,^24^,^25^,^26^. The utilisation of cell-free DNA from blood samples as a surrogate for tumour biopsy to determine EGFR mutation status represents one such promising alternative that is currently evaluated clinically to assist the diagnosis of NSCLC^15^,^16^. Here, we adopted a lipidomic-based approach to screen the PE profile so as to identify lipid differentiators suggestive of malignancy.

The combination of multivariate and univariate analyses revealed clear differences in the lipidomes between benign and malignant PE (Fig. 1). More importantly, malignant PE cases with a known EGFR mutation exhibited a more distinctive metabolic phenotype in comparison to non-EGFR mutant cases, with higher abundance of several lipid classes relative to benign PE. This observation has important implications in the identification of reliable malignancy markers that will apply to both EGFR and non-EGFR mutant cases; the commonly used strategy of biomarker identification does not take into consideration the potential heterogeneity of metabolic phenotypes between malignant cases harbouring different driver mutations (e.g. EGFR mutant vs non-EGFR mutant). As such, to account for such heterogeneity and to ensure that reliable indicators of malignancy can be selected, separate pair-wise comparisons (benign vs EGFR mutants, benign vs non-EGFR mutants) were performed. Subsequently, only lipid species which satisfied the statistical selection criteria for both sets of pair-wise comparisons were identified as candidate markers for malignancy. This strategy appears to be effective - as shown in Table 2, individual ROC analysis yielded AUC values of 0.70 and above for the majority of these lipid species, indicating that each feature had good diagnostic performance in distinguishing between benign and malignant PE samples regardless of molecular subtypes.

A large group of unsaturated or hydroxylated fatty acids were found to be elevated in our malignant PE samples. In particular, these elevations of FA(18:1) and FA(18:2) in malignancy recapitulated Lam *et al*.^20^ observations. Although our AUC values (ROC analysis) were comparatively lower to theirs (0.76–0.81 as compared to 0.962^20^), we suggest that this may be due to the different underlying medical conditions of the benign controls. In our study, benign PE samples were attributed to multiple non-malignant causes (pneumonia, tuberculosis, cardiopulmonary etc.), whereas Lam *et al*. recruited solely tuberculosis subjects as benign controls. We believe that the selection of a diversified benign patient cohort (control) would render a more robust selection of PE-derived malignancy markers as PE can be attributed to various benign causes.

In the biological context of cancer metabolism, the phenomenon of increased fatty acids and phospholipids in malignant samples may be attributed to the induction of the key lipogenic enzymes, their nuclear receptors and transcriptional regulators. Elevated abundance of fatty acids is consistent with previous reports on prostate and breast cancers^27^,^28^, and has been associated with the overexpression of fatty acid synthase (FAS). FAS overexpression is known to facilitate the *de novo* synthesis of fatty acids for the production of membrane phospholipids and energy production via beta-oxidation, and FAS is increasing being recognised as a key trait which confers tumour growth and survival advantages^29^,^30^. More specifically, the elevation of unsaturated fatty acids in our study also suggested an association with stearoyl-CoA desaturase (SCD) overexpression, which converts saturated fatty acids into unsaturated fatty acids. The observation corroborates with the reported increases in SCD-1 overexpression for both lung carcinoma cell lines^31^ and lung tumour-initiating cells^32^ in recent studies. In addition, liver X receptors (LXR) are a set of oxysterol-activated nuclear receptors involved in regulating cholesterol, fatty acid and glucose homeostasis^33^,^34^,^35^,^36^,^37^,^38^. Sterol regulatory element binding protein 1 (SREBP-1) belongs to a family of membrane-bound transcription factors that can directly activate expression of genes implicated in fatty acids, triglycerides and phospholipid metabolism^39^,^40^. Both LXR and SREBP-1 expression have been reported in human lung tissues and cell lines^41^,^42^,^43^. LXRs can regulate lipogenesis through regulating SREBP-1 or by directly targeting genes such as FAS and SCD-1 downstream of SREBP-1C pathway^33^,^34^,^38^. At the functional level, upregulation and activation of LXRs may explain the elevations of fatty acids and phospholipids. However, it is to be noted that there have been other reports of some PUFAs such as eicosapentaenoic acid (FA20:5) and docosahexaenoic acid (FA22:6) functioning as LXR antagonists^39^,^44^. As such, further mechanistic studies will likely be required to determine the detailed associations between LXR and PUFAs in NSCLC. In a recent study, Dai *et al*.^45^ found massive lung lipid accumulation, M1 macrophage-predominant lung inflammation that eventually proceed to lesions resembling peripheral squamous cell lung cancer when both LXRα and LXRβ were inactivated in LXRα; LXRβ double null mutant mice models. Together with studies illustrating the anti-proliferative effect of LXR agonists in NSCLC^45^,^46^,^47^, the findings underpin the importance of LXR signalling in lung cancer. Emerging evidence also suggest SREBP-1 is a critical link between oncogenic signalling and tumour metabolism^48^. Aberrant activation of SREBP and induction of expression of its target genes has been found in several cancer types (e.g. breast, ovarian, prostate cancer), promoting cancer proliferation, migration and invasion^49^. In certain subtypes of glioblastoma multiforme that express an activated mutant form of EGFR, high levels of nuclear SREBP-1 was observed^50^. Considering the regulatory role of EGFR-PI3K-Akt signalling to activate SREBP-1^48^,^50^ and the inherent function of SREBP-1 to regulate lipid metabolism^39^,^40^, it will be interesting to examine its association with the increase of PUFAs and phospholipids in NSCLC patients, particularly those observed in EGFR mutants in subsequent studies.

Dysregulation of sphingolipid metabolism also has important implications to cancer pathogenesis owing to their diverse structural and signalling roles in regulating cell proliferations, differentiation and apoptosis^51^. In this study, we observed elevated glycosylated ceramide and sphingomyelin species in malignancy. Ceramide, a central molecule in sphingolipid biosynthesis, is generally regarded as a powerful tumour suppressor for its ability to induce apoptosis and anti-proliferative response^51^. Conversely, glycosylation of ceramide by glucosylceramide synthase (GCS) into glucosylceramide and glycosphingolipids is known to stimulate tumour cell proliferation, inhibit apoptosis and promotes tumour cell survival^51^. Further, increase GCS activities have been implicated in the development of chemotherapy resistance in several types of cancer^52^,^53^. Additionally, ceramide levels can also be attenuated through the activity of sphingomyelin synthase (SMS) that transfers phosphocholine headgroup to ceramide forming sphingomyelin. The functions of SMS may however go beyond the regulation of ceramide levels. For instance, sphingomyelin isolated from shed membrane vesicles of tumour cells has been demonstrated to promote tumour growth, metastases, invasiveness, endothelial cell migration and angiogenesis^54^. Interestingly, a comparison of human lung tumour tissues compared to their matched non-tumour tissues revealed lower SMS-1 expression^55^ and sphingomyelin abundance in lung tumour tissues^56^. SMS activation by anti-tumour 2-hydroxyoleic acid to A549 human lung adenocarcinoma cells has been found to increase SM at plasma membrane, modify lipid raft properties, enhance localization of protein involved in cell apoptosis or differentiation and trigger tumour cell death^57^. On the other hand, ceramide-enriched rafts, generated from the displacement of cholesterol from the raft by membrane ceramide released from sphingomyelin, has been proposed to lead to re-localization of EGFR in the ceramide-enriched rafts, thereby stabilizing aberrant EGFR signalling^58^,^59^,^60^,^61^,^62^,^63^. As such, the intricate balance between these sphingolipids is crucial in determining the fate of cancer cells due to their biologically active roles. Our identification of elevated glycosylated ceramide and sphingomyelin species in the malignant PE may hence reflect the lung cancer cells’ attempt to regulate sphingolipid metabolism to neutralize the downstream cell death signals initiated by ceramide.

In our study, we observed a more distinctive metabolic phenotype for the EGFR mutants, characterised by trends of higher relative fold change and higher AUC values for the pair-wise comparison between benign and EGFR mutant cases in comparison to non-EGFR mutants. As such, we further scrutinize for differential lipids between EGFR mutants and non-EGFR mutants [satisfying the selection criteria for both pairwise comparisons (EGFR mutant vs benign cases, EGFR mutant vs non-EGFR mutant cases)] (Table 3). These lipid species belonged to two major lipid classes, including PUFAs and phospholipids. PUFAs have been previously implicated in the activation and phosphorylation of EGFR in human endothelial cells^64^. More significantly, the same study reported that the EGFR-phosphorylating activity of fatty acids was associated with chain length and degree of saturation. PUFAs with longer chain lengths such as FA(20:5) and FA(22:6) were more effective in activating EGFR, while short chain, saturated fatty acids had no effect on the receptor. These results corroborated with the fatty acid species we have identified, which were comprised solely of 20 and 22 chain length PUFAs. Currently, the protective roles of PUFAs in cancer development remain controversial^65^,^66^,^67^. Our observation revealed a positive association of higher PUFAs levels in PE of EGFR mutants. As it is possible that PUFAs in EGFR mutant cells might play a role in sustaining the higher EGFR activation rate, the mechanistic function of PUFAs in EGFR and non-EGFR driven NSCLC should be further elucidated to determine its effect on the growth inhibition or promotion of EGFR mutant tumours^64^,^68^. Nonetheless, the biological relevance of these fatty acids substantiated their roles as biomarkers for diagnosis and patient stratification as illustrated by their diagnostic performance (e.g. AUC and ACC).

Long chain and ether-linked PEtn species, including lysoPEtn(P-16:0), were another lipid class in differential abundance between EGFR mutants and non-EGFR mutants. Ether-linked species, also known as plasmalogens, are reported to play pertinent physiological roles in intracellular signalling and confer protective roles during oxidant-induced stress^69^. Elevated levels of plasmalogens have previously been found in various cancer tumours^70^, as well as breast and melanoma cancer cell line models^71^. In the same study, the authors reported the heightened expression of alkylglyceronephosphate synthase (AGPS), a critical enzyme for the synthesis of ether-linked lipids, in both the cell lines and primary breast tumours. Subsequent knockdown of AGPS resulted in the impairment of migration, invasion, cell proliferation and survival in both cell lines. In our study, plasmalogen species appear to be generally higher in EGFR mutants when compared to both non-EGFR mutants and benign PE samples (Fig. 1C). This lipid signature suggests that a more active ether-linked lipid synthesis pathway might be present in EGFR mutants and the plausible role of AGPS in the pathogenesis of EGFR mutation-driven NSCLC remains to be determined.

Based on our ROC analysis of the lipid markers identified in Tables 2 and 3, the lipid species displayed good performance diagnostics (e.g. AUC, SN, SP and ACC) when used singly. Using SVM, we selected an optimal number of lipid markers for use in combination from the list of 45 candidate malignancy markers in the heat map in Fig. 1C. By combining the lipid markers, we were able to achieve improvement in the diagnostic performance to stratify patients based on their PE lipid composition into the following groups: (i) benign vs malignant and (ii) EGFR vs non-EGFR mutants. Some of the lipid species such as FA(22:5), FA(22:6), FA(20:5) were identified as candidate diagnostic markers by both the pairwise comparison strategy and the SVM strategy, to be used either singly or in combination. This underscored their importance in NSCLC and/or the underlying driver mutation. Interesting, the SVM modelling also unveiled other lipids species (Figs 2C and 3E) that contributed towards the stratification of the patients not identified using our criteria (Tables 2 and 3) which deserves further investigations.

There are specific strengths and limitations that have to be considered for this study. Using a global lipidomics approach, we were able to detect a broad range of structurally diverse lipid species and elucidate interesting and important biomarkers for the diagnosis of NSCLC that is currently unexplored. As PE can occur due to a variety of non-malignant causes, we recruited subjects admitted due to a wide range of underlying pathology (e.g. pneumonia, tuberculosis and cardiopulmonary congestion) as “benign” controls so as to identify biomarkers that can be used in a wide clinical context. In consideration of the unique metabotype of NSCLC patients with different molecular subtypes (EGFR- and non-EGFR mutants), we adopted a pairwise comparison approach to screen for lipid malignancy markers with good diagnostic performance instead of treating them as a homogenous group. A limitation, however, was that the small sample size for other driver mutations did not allow for the derivation of additional unique lipidomic signatures which correlated to their respective genotypes to be performed. The semi-quantitative nature associated with such untargeted studies also calls for follow-up targeted studies involving an independent validation cohort comprising of a larger number of patients, to verify the predictive accuracy of these novel candidate markers and their ability to complement cytological assessment of PE samples. Additionally, mechanistic validation studies may also be performed on EGFR mutant and non-EGFR mutant cell lines to elucidate the biological roles of these candidate malignancy lipids, as this will provide interesting mechanistic insights to substantiate their relevance in NSCLC.

In conclusion, the study yielded a series of lipid molecules, many of which are novel, that show promise as potential malignancy and/or EGFR mutant specific markers. In particular, 20 and 22 carbon chain length PUFAs had the highest potential in distinguishing between benign, EGFR mutant and non-EGFR mutant PE samples. Such biomarkers will not only facilitate diagnosis, but also assist in patient stratification for personalised therapy (e.g. administration of TKIs to patients with EGFR mutation). The use of SVM modelling was found to facilitate the selection of an optimal combination of biomarkers that showed improved diagnostic performance compared to using single biomarkers. Additionally, our work highlights the importance of taking different phenotypic behaviour of different genotypes into consideration in the selection of suitable malignancy candidate markers. Finally, information on the perturbation of lipid pathways in NSCLC gleaned from lipidomic profiling has shed new and established insights that further substantiate the importance of lipid metabolic reprogramming in cancer biology.

## Patients and Methods

### Study Patient and Sample Collection

Lung PE samples were obtained from 71 patients admitted to Singapore General Hospital and National Cancer Centre Singapore between December 2012 and December 2014. These PE samples collected via thoracocentesis were centrifuged at 805 *g* at 4 °C for 10 mins upon collection. The NSCLC malignant cases were assessed based on clinical diagnosis, cytology of the cells isolated from PE and histology of tumour tissues. Patients whose histological examinations did not show any malignancy were classified as benign subjects. The patient demographics and characteristics, including age, gender, cytology and histology are provided in Table 1. In our study, there were 30 benign and 41 malignant cases. Of the malignant cases, 19 cases were confirmed to harbour EGFR mutations (EGFR mutant), 17 cases did not possess EGFR mutations (non-EGFR mutant), while the molecular status of the remaining 5 malignant cases were unknown due to insufficient samples for mutation testing following cytology and histology examination. All the PE supernatants were stored at −80 °C prior to LC-MS-based lipidomic analysis. All patients recruited gave informed consent and this study was approved by and carried out in accordance with the guidelines of the Singapore Health Services centralized institutional review board (CIRB 2010/516/B).

### Reagents and Materials

Reagents were obtained as follows: Optima grade methanol, isopropanol: Fisher Scientific (Loughborough, UK); tricine: Sigma-Aldrich (St Louis, MO); ammonia solution “AnalaR” 25%: VWR (Poole, UK); acetonitrile, chloroform, acetic acid, formic acid, ^13^C-labelled isotopic fatty acid standards (palmitoleic acid-^13^C_16_, palmitic acid-1,2,3,4-^13^C_4_, linoleic acid-^13^C_18_, stearic acid-^13^C_18_): Merck (Whitehouse Station, NJ); odd-chain lipid standards: phosphatidylcholine, PC(9:0/9:0); PC(17:0/17:0); PC(21:0/21:0); phosphatidylethanolamine, PEtn(15:0/15:0); PEtn(17:0/17:0): Avanti Polar Lipids (Alabaster, AL). The ^13^C-labelled isotopic and odd-chain lipid standards constitute the lipid reference standard mixture.

### Lipid Extraction

50 μL of each PE sample was aliquoted and 10 μL of lipid reference standard mixture was added to the sample as retention time reference prior to a two-phase modified Bligh and Dyer^72^ lipid extraction protocol. Each sample was extracted by sequential addition of methanol, chloroform and 3.8 mM tricine (1:1:0.5 v/v/v, total 2 mL), with sample vortexed for 1 min following each addition. The samples were then centrifuged at 12,000 *g* at 4 °C for 20 min, following which each sample separated into two fractions–the top methanolic layer contained the polar metabolites while the bottom chloroform layer contained the lipid species. The bottom chloroform fraction enriched with lipid species was collected and stored at −80 °C prior to analysis. Quality control (QC) samples were prepared by mixing equal amount of all the PE samples and each QC sample was extracted as described above. All samples were randomized for extraction.

### Lipidomic Profiling using LC-MS

An ultra-high performance liquid chromatography (UHPLC) system (Ultimate 3000, ThermoFisher Scientific, MA) interfaced with a Q-Exactive mass spectrometer (ThermoFisher Scientific, MA) was utilized for lipidomic analysis. Prior to each analytical batch, instrument maintenance (source cleaning and mass calibration) was performed. A reversed phase LC column (Acquity CSH, 1.0 × 50 mm, 1.7 μm particle size, Waters Corp) was used for separation with two solvents: ‘A’ comprising of acetonitrile, methanol and water (2:2:1) with 0.1% acetic acid and 0.1% ammonia solution, and ‘B’ comprising of isopropanol with 0.1% acetic acid and 0.1% ammonia solution. All samples were dried using a sample concentrator (Bio-techne, Minneapolis, MN), reconstituted in a 50:50 (v/v) mixture of solvents A and B. The UHPLC autosampler temperature was set at 4 °C and the injection volume for each sample was 2 μL. All samples were processed in technical triplicates on the LC-MS system. The LC program is as follows: the column was first equilibrated for 1 min at 1% B with a flow rate of 0.1 ml min^−1^. The gradient was increased from 1% B to 82.5% B over 9 min before B was increased to 99% for a 5 min wash at a flow rate of 0.15 ml min^−1^. The column was re-equilibrated for 2.2 min at 1% B. Column temperature was maintained at 45 °C. The eluent from the LC system was directed into the MS. Electrospray ionization (ESI) in the MS was conducted in both positive and negative modes in full scan with a mass range of 120 to 1800 m/z, resolution of 70,000, automatic gain control (AGC) target of 1 × 10^6^ ions (ESI+) or 3 × 10^6^ ions (ESI−) and maximum injection time (IT) of 100 ms (ESI+) or 200 ms (ESI−). The heated electrospray ionization (HESI) source used a spray voltage of 3.7 kV (ESI+) and 3.2 kV (ESI−), capillary temperature of 350 °C (ESI+ and ESI−), sheath gas flow of 25 (ESI+ and ESI−) and auxiliary gas flow of 10 arbitrary units (ESI+ and ESI−).

QC samples were analysed at regular intervals throughout each batch analysis to monitor the reproducibility of the LC-MS. The extracted samples were re-randomized for LC-MS analysis such that the injection order was independent from the order of sample preparation to minimise systematic bias.

### Data Pre-processing and Statistical Analysis

The raw LC-MS data obtained was then pre-processed and analysed using the XCMS peak finding algorithm^73^. The spiked lipid reference standards had relative standard deviations of less than 20% across all samples, demonstrating the high reproducibility of our extraction and LC-MS method. The QC mixture was used for signal correction between and within each batch analysis. Mass peaks with poor repeatability within the QC samples (coefficient of variation more than 30%) were removed. Total area normalisation (based on ratio of area of each mass peak against sum of peak areas within each sample) was applied to the remaining features in the dataset to correct for minor variations in sample preparation and analysis. The normalised data were exported to SIMCA-P+ (version 13.0.3, Umetrics, Umea, Sweden) for multivariate data analysis to identify potential PE biomarkers.

Data were mean-centred and Pareto scaled in SIMCA-P+. Subsequently, an unsupervised principal component analysis (PCA) was utilised to determine the quality of LC-MS data obtained based on the tight clustering of the QC samples and to derive an overview of similarities and differences between individual PE samples. This was followed by supervised orthogonal projection to latent structures-discriminant analysis (OPLS-DA) where a model was built to identify individual lipid components that were distinctly different between (1) benign, (2) EGFR mutant and (3) non-EGFG mutant malignant cases in a pair-wise manner. These lipid components are identified based on their variable importance for projection (VIP) values. Lipid species with higher VIP (VIP > 1) made a greater contribution towards distinguishing the comparator groups in the OPLS-DA model and were considered as potential biomarkers. Univariate analysis was performed using the Mann-Whitney U test at p-value < 0.05 to verify the statistical significance of these potential biomarkers. Fold change was calculated by taking the ratio of the peak areas contributed by the lipid species of the two comparator groups.

### Support Vector Machines Modelling

The support vector machines (SVMs) model, first proposed by Vapnik and his colleagues^74^, is a widely used machine learning technique for pattern recognition. The SVMs construct a boundary that maximizes the distance between the designated class of each sample (e.g. whether the sample is “benign” or “malignant”). An optimal boundary separating the sample class is then defined. In this study, we used the popular libSVM package^75^ with linear kernel function to perform the classification, where involved parameters are automatically selected by Bayesian Optimization^76^. The recursive feature elimination (RFE) method, based on backward sequential selection strategy^77^, was used to select the best features of the SVM classifier. Starting with a full candidate set of malignancy lipid markers, features (lipid markers) were removed sequentially such that the variation of separating boundary was minimized and until the desired number of features was reached. Different desired number of features was evaluated to determine the performance of the various feature combinations.

In the construction of a real pattern classification system, the data available are generally limited, such that there is a need for a validation technique to estimate how a classification system will perform in practice. In our study, the k-fold cross-validation^78^ is used to estimate the classification performance. In a single round of k-fold cross-validation, the dataset is first randomly portioned into k subsets (folds), which are of approximately equal size and are mutually exclusive. A SVM classifier is then trained and tested k times, and at each time, one of the subsets is set aside as the testing data while the remaining k − 1 subsets set as the training data. In our study, leave-one-out cross validation (i.e. k = 71) was used.

ROC analyses were then performed for the two optimal combinations of lipid markers capable of differentiating the PE between (i) the benign and malignant patients and (ii) non-EGFR and EGFR mutants. ROC analyses were also performed for the identified lipid species (VIP > 1, p-value < 0.05, fold change ≥1.5). The ROC is plotted using Stata/MP 14.0 statistical package (Stata Corp, LP) based on the predicted real value of each sample from the trained SVM model.

### Metabolite Identification

Mass peaks were first putatively identified based on mass comparison (less than 5 ppm error) with entries from the Kyoto Encyclopedia of Genes and Genome (www.genome.jp/kegg) and the Human Metabolome Database (www.hmdb.ca). Subsequently, the identities of lipid species of interest were verified by MS^2^ spectral comparison with commercially available standards where possible, or by comparison to mass spectral databases available online.

## Additional Information

**How to cite this article**: Ho, Y. S. *et al*. Lipidomic Profiling of Lung Pleural Effusion Identifies Unique Metabotype for EGFR Mutants in Non-Small Cell Lung Cancer. *Sci. Rep.* **6**, 35110; doi: 10.1038/srep35110 (2016).

## Supplementary Material

Supplementary Information

## Figures and Tables

**Table 1:** Clinical characteristics of benign subjects (n = 30) and malignant non-small cell lung cancer patients (n = 41).

Characteristic	All Patients (n = 71)	Benign (n = 30)	Malignant (n = 41)
Gender			
Male, %	35 (49.3)	18 (60.0)	17 (41.5)
Female, %	36 (50.7)	12 (40.0)	24 (58.5)
Age at sample collection, years			
Mean ± standard deviation	67 ± 14	63 ± 16	69 ± 12
Smoking status			
Smoker (Current/Ex)	25	9	16
Non smoker	46	21	25
Ethnic group			
Chinese, %	61 (86.0)	25 (83.3)	36 (87.8)
Malay, %	5 (7.0)	1 (3.3)	4 (9.8)
Indian, %	4 (5.6)	3 (10.0)	1 (2.4)
Others, %	1 (1.4)	1 (3.3)	0 (0.0)
Histology			
Non-small cell lung adenocarcinoma	39 (54.9)		
Squamous-cell carcinoma	1 (1.4)		
Lymphoepithelioma-like lung carcinoma	1 (1.4)		
Non-malignant	30 (42.3)		
Cytology assessment			
Positive for malignancy	32 (45.0)		
Negative for malignancy	30 (42.3)		
Suspicious/atypical confirmed as malignant based on histology	9 (12.7)		
Mutation subtypes for malignant cases			
EGFR +			19 (46.3)
EGFR −		N.A.	17 (41.5)
Unknown			5 (12.2)
Subtypes for non-malignant cases			
Pneumonia		22 (73.3)	
Cardiopulmonary congestion		5 (16.7)	N.A.
Tuberculosis		3 (10.0)	

n, Number of cases; EGFR, Epidermal growth factor receptor.

**Table 2:** Potential lipid malignancy markers for differentiating the PE of benign and malignant patients with NSCLC and their diagnostic performance.

Lipid name^a^	Benign vs non-EGFR mutants	Benign vs EGFR mutants	Diagnostic performance as malignancy marker			
	Ratio^b^	Ratio^b^	AUC^c^	SN (%)	SP (%)	ACC (%)
*Unsaturated/hydroxylated fatty acids*						
Hydroxyl FA(16:0) *[(R)-2-Hydroxyhexadecanoic acid]*	1.56	1.83	0.74 (0.62–0.85)	73.17	63.33	69.01
FA(14:2) *[5,8-Tetradecadienoic acid]*	1.64	2.54	0.77 (0.65–0.88)	78.05	70.00	74.65
FA(18:1) *[Oleic acid]*	1.80	1.71	0.76 (0.65–0.88)	70.73	73.33	71.83
FA(18:2) *[Linoleic acid]*	1.65	1.95	0.81 (0.70–0.91)	80.49	76.67	78.87
FA(18:3) *[Linolenic acid]*	1.53	1.81	0.74 (0.63–0.86)	70.73	70.00	70.42
FA(20:5) *[Eicosapentaenoic acid]*	1.82	4.43	0.79 (0.68–0.90)	75.61	73.33	74.65
FA(22:4) *[Adrenic acid]*	2.20	2.33	0.80 (0.69–0.90)	75.61	73.33	74.65
FA(22:5) *[Docosapentaenoic acid]*	2.46	6.11	0.87 (0.79–0.96)	82.93	83.33	83.10
FA(22:6) *[Docosahexaenoic acid]*	1.89	3.17	0.87 (0.79–0.95)	82.93	83.33	83.10
*Sphingolipids*						
GalCer(d40:1)/GlcCer(d40:1)	1.82	1.51	0.72 (0.60–0.85)	80.49	60.00	71.83
SM(d44:1)	1.53	1.53	0.73 (0.62–0.85)	63.33	69.01	64.79
SM(d42:2)	1.64	1.74	0.66 (0.54–0.79)	63.33	64.79	69.01

^a^Individual abbreviated lipid names are provided based on the following convention: Lipid class (total number of carbons: total number of double bonds).

^b^Ratio calculated relative to benign.

^c^AUC value obtained based on receiver operating characteristic (ROC) analysis with 95% confidence interval range provided in parentheses.

VIP, variable importance for projection value; AUC, area under curve for ROC analysis; SN, sensitivity; SP, specificity; ACC, accuracy; FA, fatty acid; GalCer/GlcCer, galactosylceramide/glucosylceramide; SM, sphingomyelin. Listed markers satisfy statistical threshold (ratio > 1.5, p-value < 0.05, VIP > 1) in both pair-wise comparisons between EGFR (n = 19) and non-EGFR mutant (n = 17) cases with benign PE (n = 30).

**Table 3:** Lipid candidates capable of distinguishing EGFR mutation status in the PE of NSCLC patients and their diagnostic performance.

Non-EGFR vs EGFR mutants					
Lipid name^a^	Ratio (relative to non-EGFR)	AUC^b^	SN (%)	SP (%)	ACC (%)
*Polyunsaturated fatty acids*					
FA(20:3) *[Eicosatrienoic acid]*	1.67	0.68 (0.50–0.87)	68.42	70.59	69.44
FA(20:5) *[Eicosapentaenoic acid]*	2.43	0.68 (0.50–0.87)	89.47	58.82	75.00
FA(22:5) *[Docosapentaenoic acid]*	2.49	0.78 (0.62–0.94)	73.68	70.59	72.22
FA(22:6) *[Docosahexaenoic acid]*	1.68	0.75 (0.58–0.93)	89.47	64.71	77.78
*Phospholipids*					
LysoPEtn(P-16:0)	1.57	0.70 (0.52–0.88)	73.68	70.59	72.22
PC(41:6)	2.60	0.70 (0.51–0.88)	63.16	82.35	72.22
PEtn(P-36:5)	2.30	0.67 (0.48–0.85)	63.16	70.59	66.67

^a^Individual abbreviated lipid names are provided based on the following convention: Lipid class (total number of carbons: total number of double bonds).

^b^AUC value obtained based on receiver operating characteristic (ROC) analysis with 95% confidence interval range provided in parentheses.

VIP, variable importance for projection value; AUC, area under curve for ROC analysis; SN, sensitivity; SP, specificity; ACC, accuracy; FA, fatty acid; LysoPEtn(P-), ether-linked lysophosphatidylethanolamine; PC, phosphatidylcholine; PEtn(P-), ether-linked phosphatidylethanolamine. Listed markers satisfy statistical threshold (ratio > 1.5, p-value < 0.05, VIP > 1) in both pair-wise comparisons of EGFR mutant cases with benign PE and non-EGFR mutant cases.
