# Supplementary material for: Lipidomic Profiling of Lung Pleural Effusion Identifies Unique Metabotype for EGFR Mutants in Non-Small Cell Lung Cancer
Source: Sci Rep. 2016 Oct 14;6:35110. doi: 10.1038/srep35110 (PMC5064315; doi:10.1038/srep35110)
Supplement: Supplementary Information [file srep35110-s1.pdf]

Supplementary Materials for

Lipidomic Profiling of Lung Pleural Effusion Identifies Unique Metabotype for EGFR Mutants in Non-Small Cell Lung Cancer

Ying Swan Ho[#*], Lian Yee Yip[#], Nurhidayah Basri, Vivian Su Hui Chong, Chin Chye Teo, Eddy Tan, Kah Ling Lim, Gek San Tan, Xulei Yang, Si Yong Yeo, Mariko Si Yue Koh, Anantham Devanand, Angela Takano, Eng Huat Tan, Daniel Shao Weng Tan[*], Tony Kiat Hon Lim[*]

[#] these authors contributed equally to this work

*Correspondence to: ho_ying_swan@bti.a-star.edu.sg

This file includes:

Figure S1: Extracted ion chromatogram (XIC) and exemplified MS/MS spectra of lipids species in the (A) ceramide, (B) lysophospholipid, (C) phosphatidylethanolamine, (D) phosphatidylcholine and (E) acylcarnitine lipid classes.

Figure S2: KEGG human metabolic pathway map of exemplified perturbed lipid pathways in which the differential lipid metabolites derived from individual pairwise comparisons between benign, non-EGFR mutant and EGFR mutant PE samples presented in Figure 1C are found.

Table S1: Exemplified list of lipid species identified in human pleural effusion

Figure S1

A. Ceramide

XIC of Gb3(d42:2), Trihexosylceramide(d18:1/24:1)

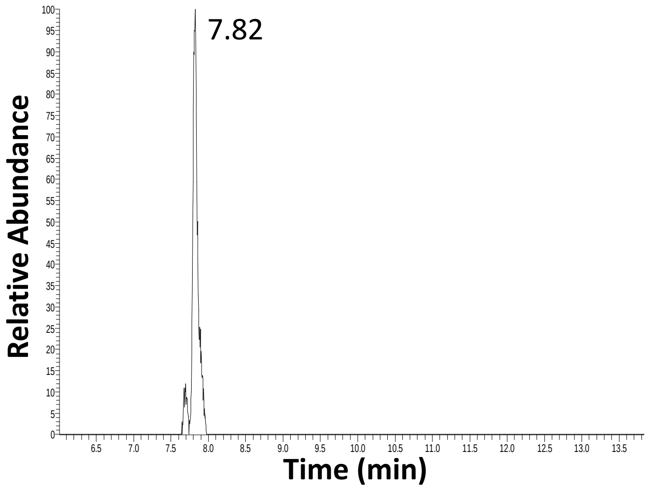

MSMS spectrum of Gb3(d42:2) in ESI+ mode

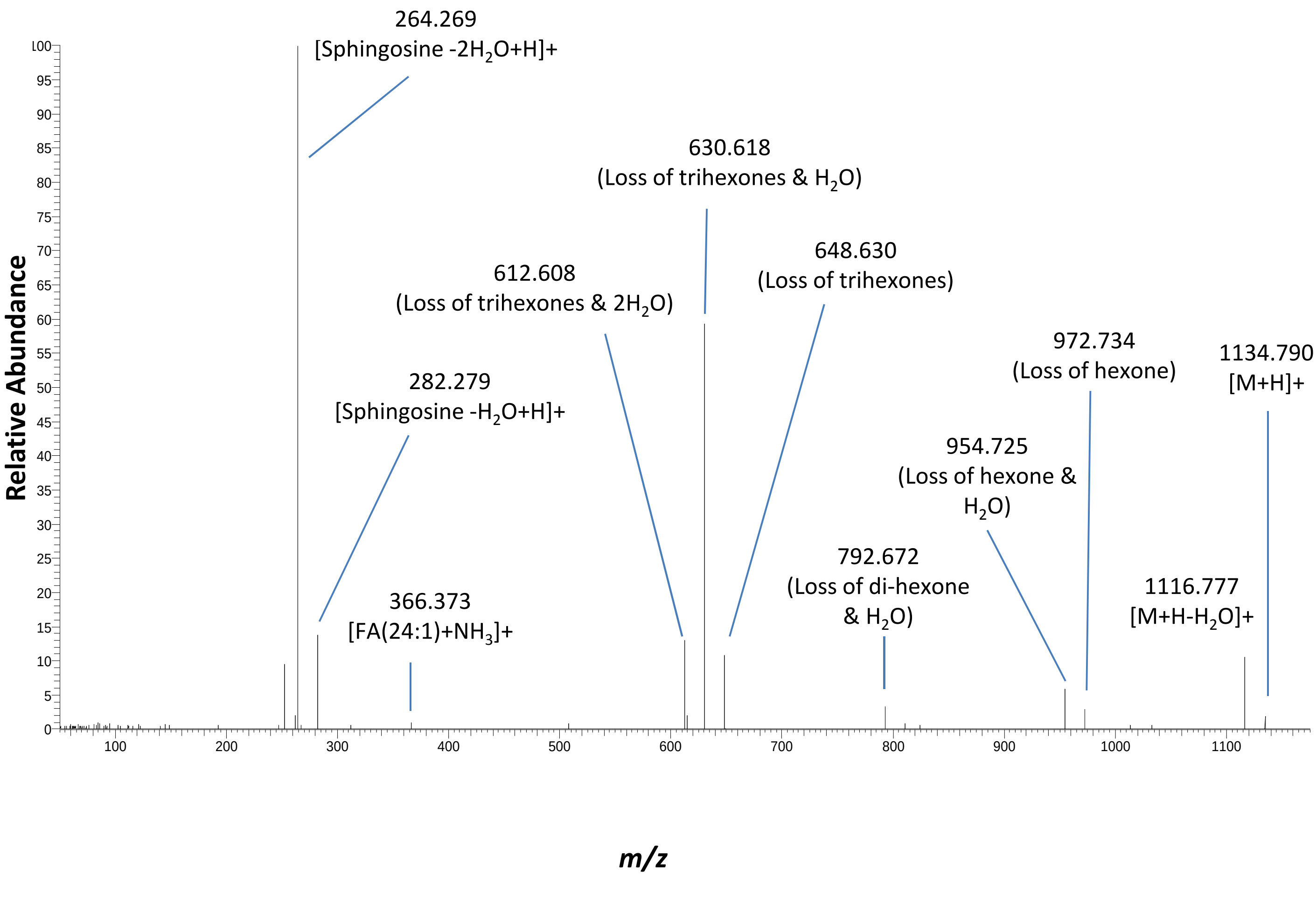

Figure S1

B. Lysophospholipid

XIC of LysoPC(22:6)

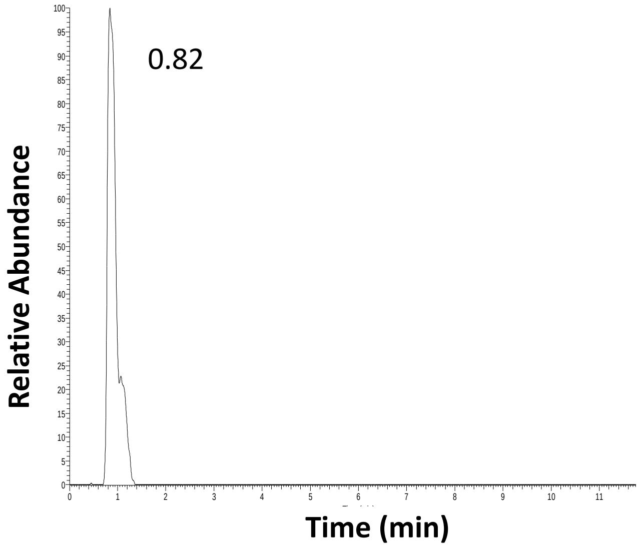

MSMS spectrum of LysoPC(22:6) in ESI+ mode

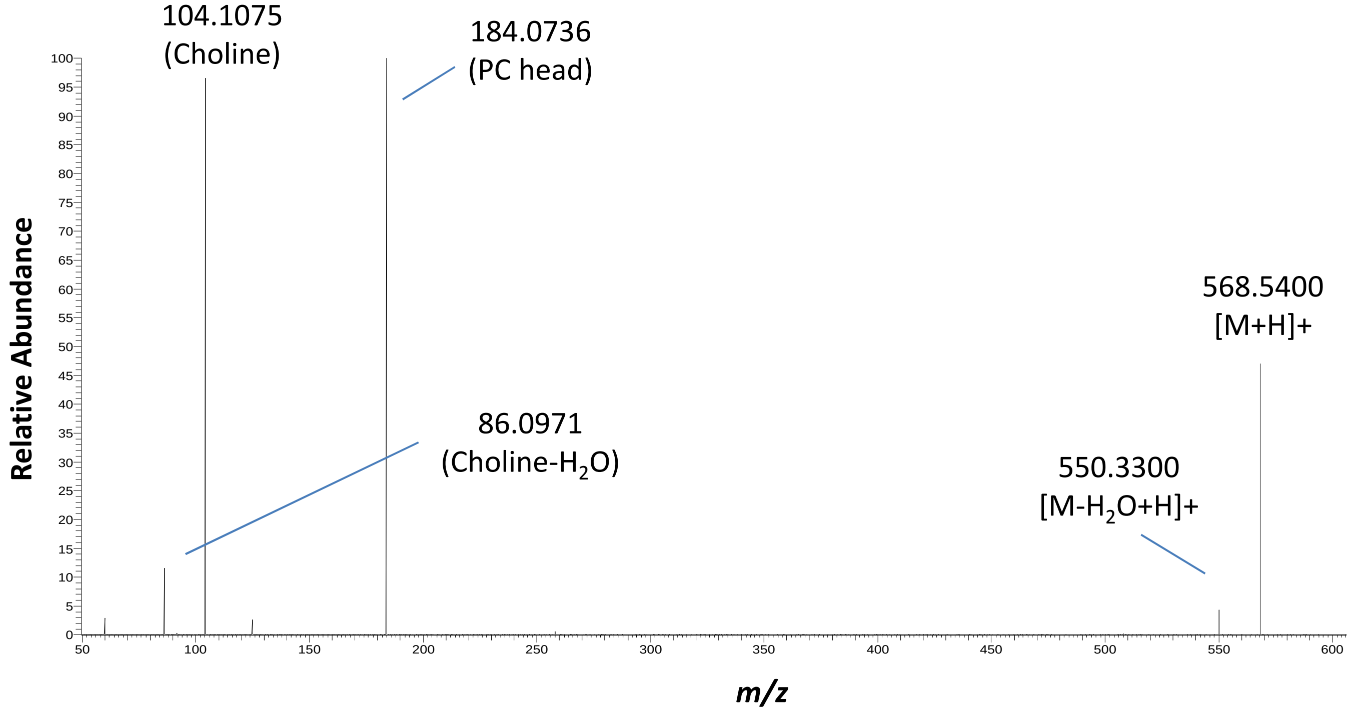

Figure S1

C. Phosphatidylethanolamine

XIC of PEtn(38:4); PEtn(18:0/20:4)

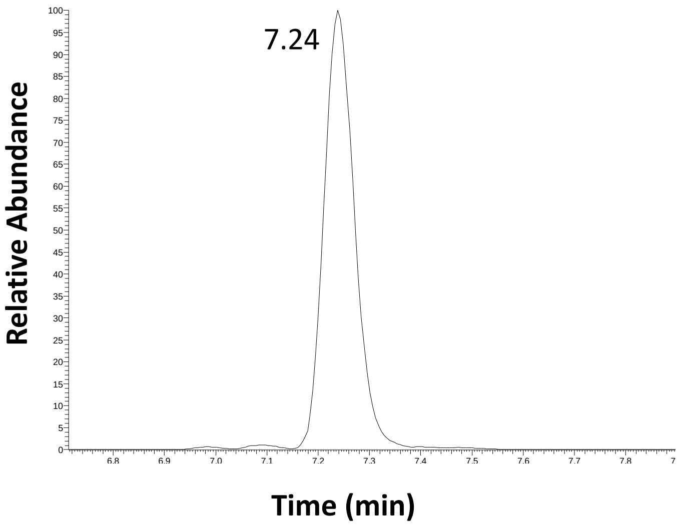

MSMS spectrum of PEtn(38:4) in ESI- mode

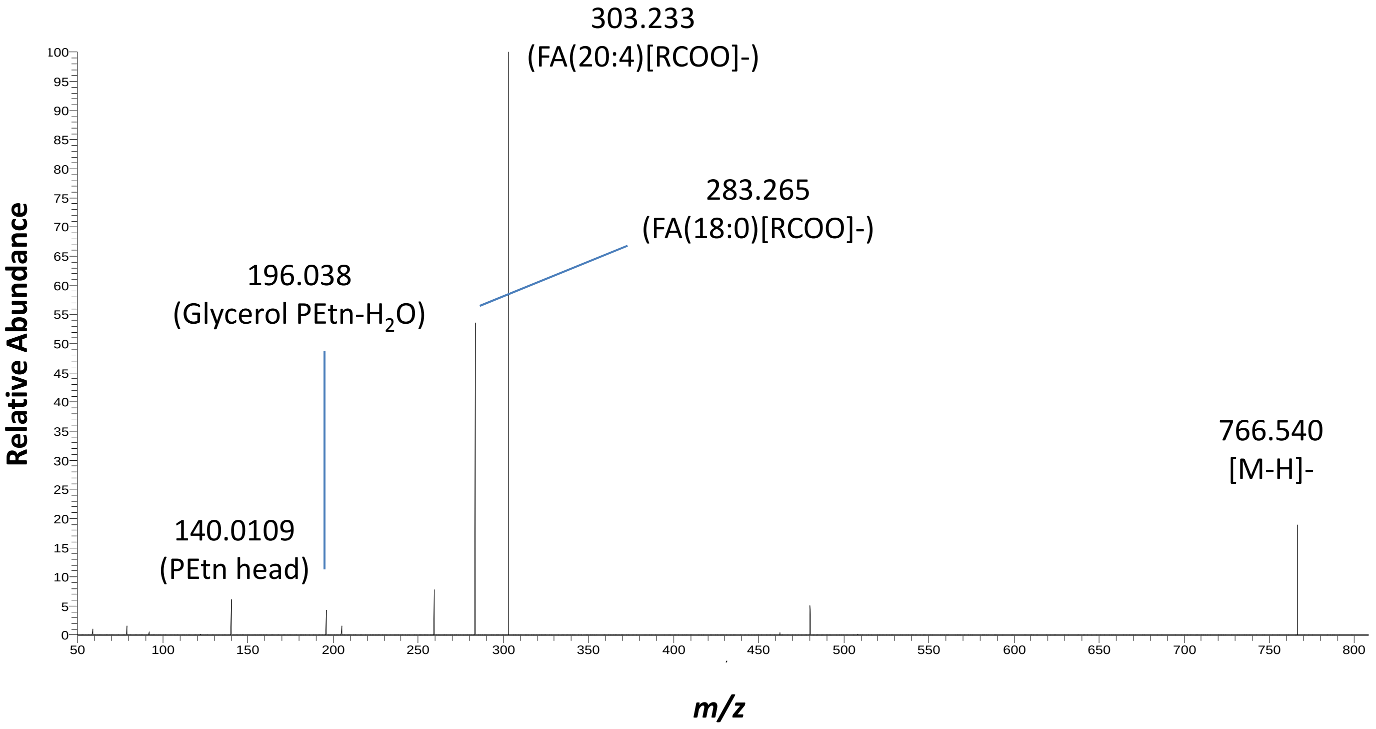

Figure S1

D. Phosphatidylcholine

XIC of PC(34:2); PC(16:0/18:2)

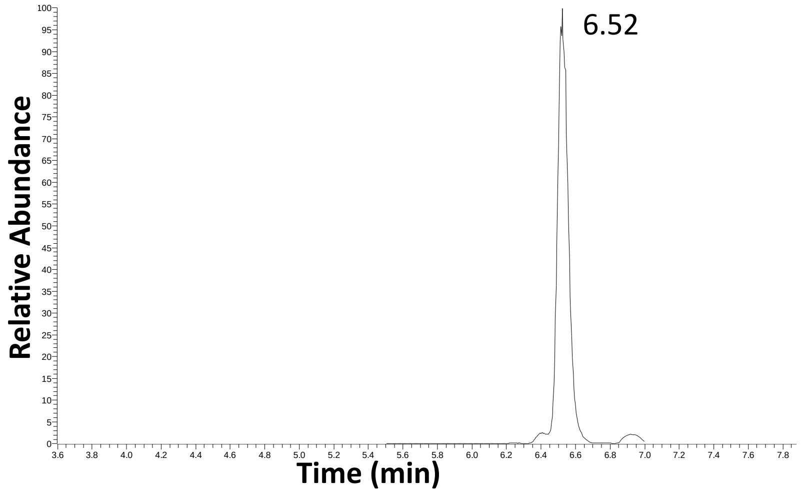

MSMS spectrum of PC(34:2) in ESI+ mode

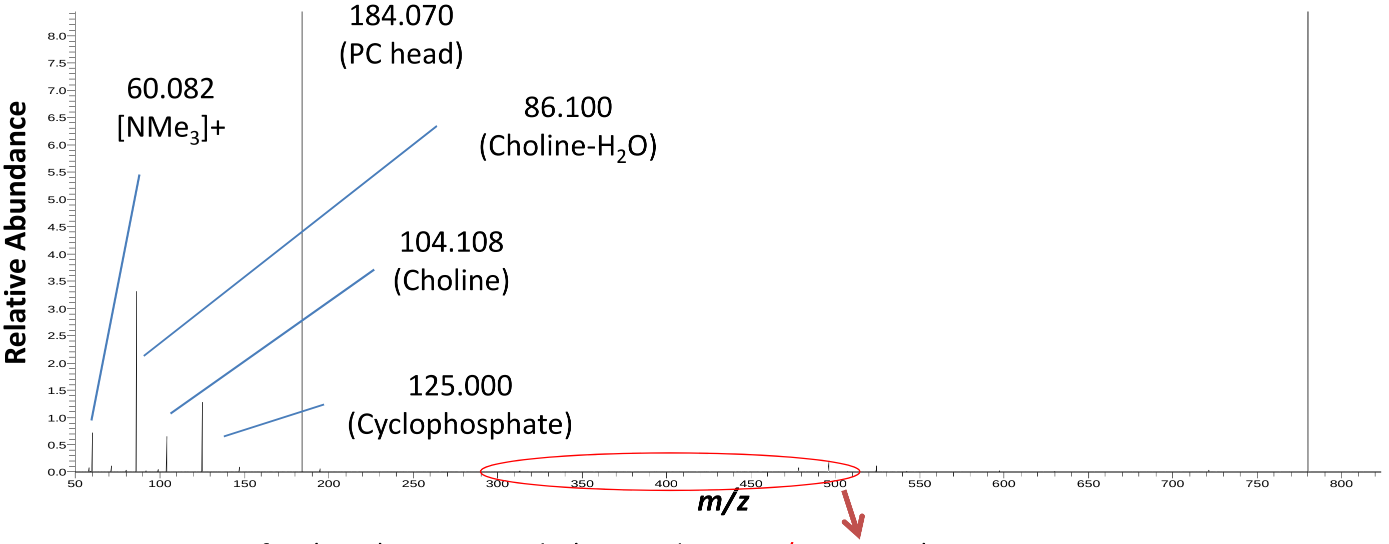

MSMS spectrum of PC(34:2) in ESI+ mode (zoomed in at m/z 300-500)

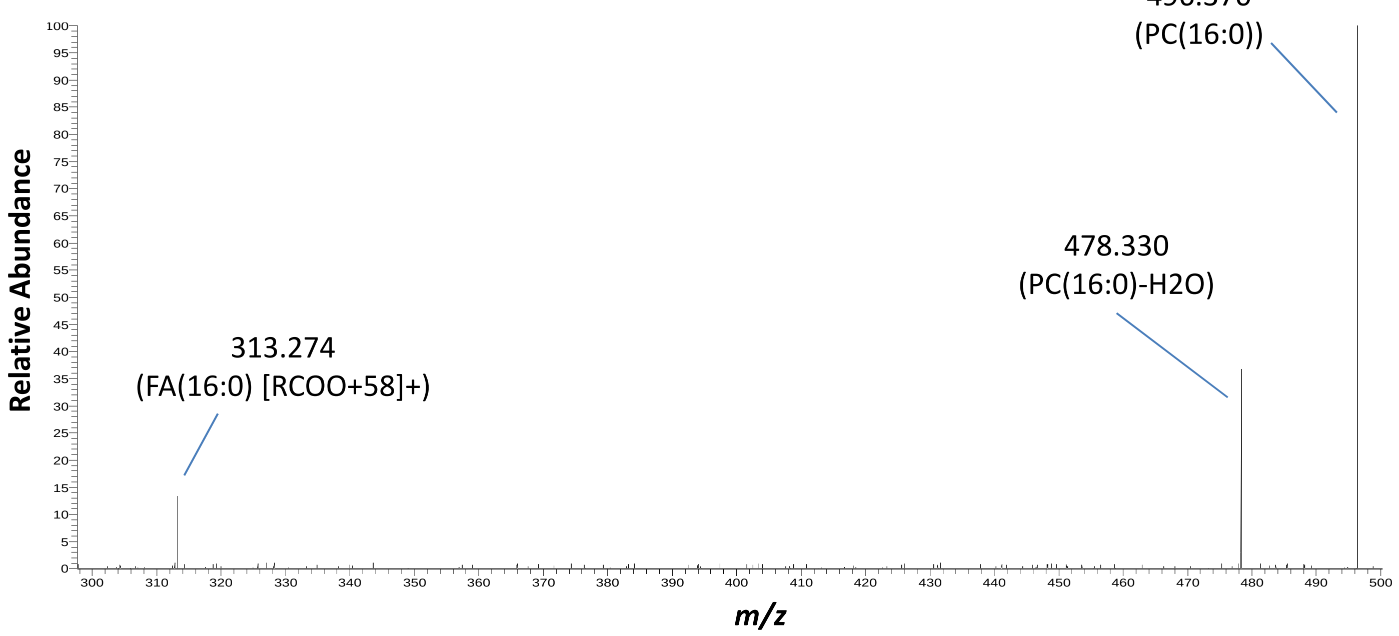

Figure S1

E. Acylcarnitine

XIC of AcylCar(18:2);Linoelaidyl carnitine

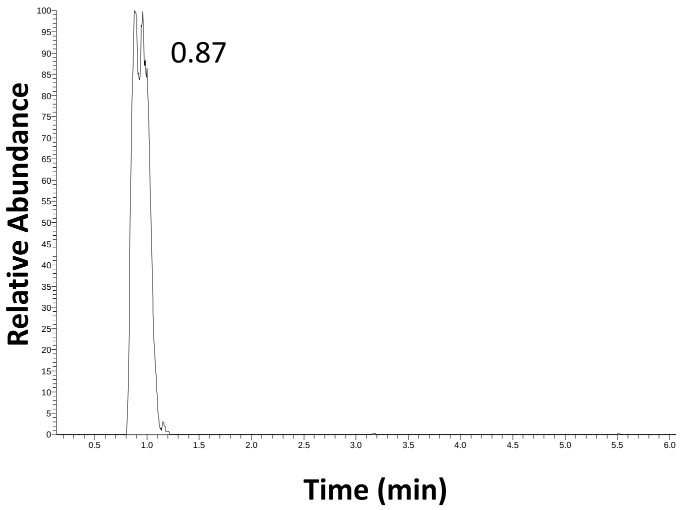

MSMS spectrum of AcylCar(18:2) in ESI+ mode

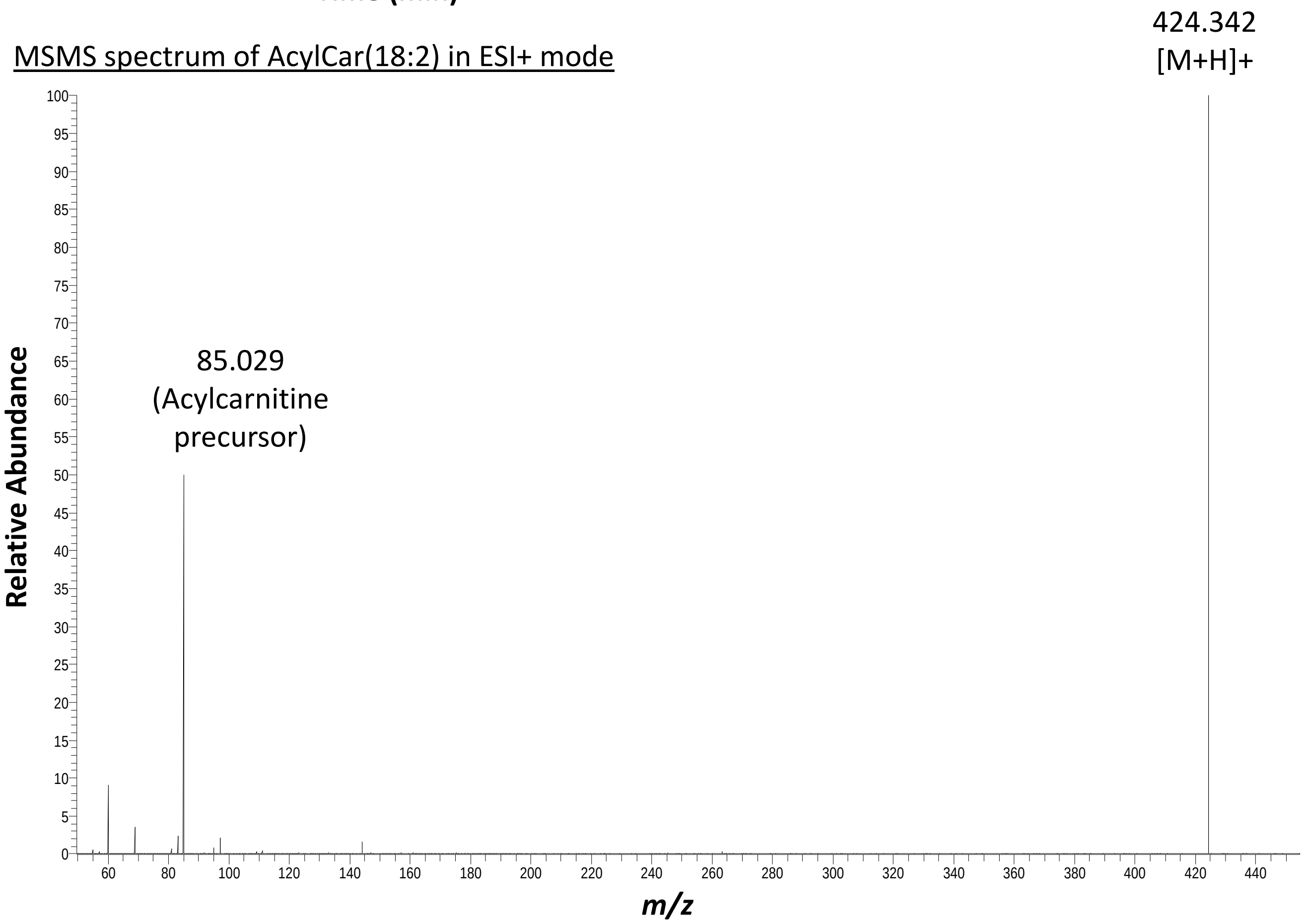

Figure S2

Linoleic acid metabolism

1. Phosphatidylcholine
   - PC(34:2)
   - PC(36:5)
   - PC(40:8)
   - PC(41:6)
2. Arachidonic acid
3. Linoleic acid
4. Linolenic acid

Glycerophospholipid metabolism

1. Phosphatidylcholine
   - PC(34:2)
   - PC(36:5)
   - PC(40:8)
   - PC(41:6)
2. Phosphatidylethanolamine
   - PEtn(34:1)
   - PEtn(36:4)
   - PEtn(38:4)
3. 1-Acyl-sn-glycero-3-phosphocholine
   - LysoPC(22:6)

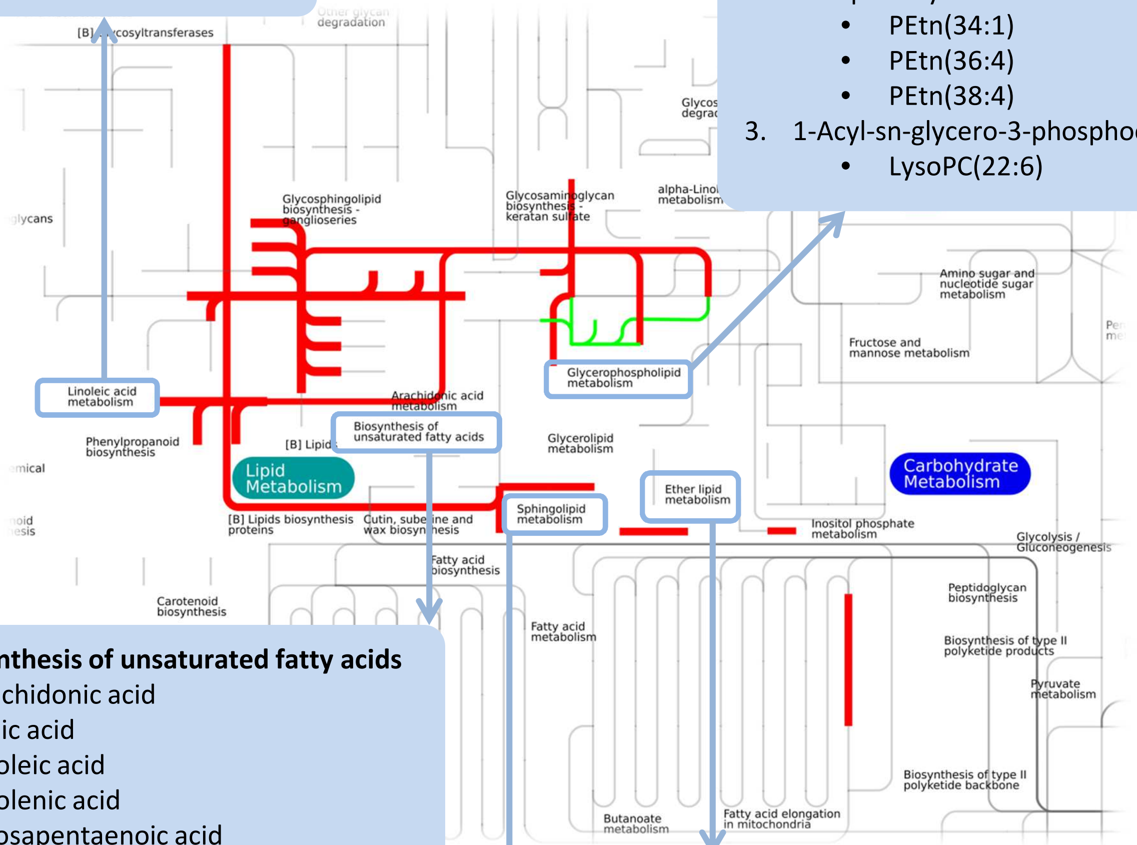

Biosynthesis of unsaturated fatty acids

1. Arachidonic acid
2. Oleic acid
3. Linoleic acid
4. Linolenic acid
5. Eicosapentaenoic acid
6. Docosahexaenoic acid
7. Docosapentaenoic acid
8. Eicosatrienoic acid
9. Adrenic acid

Sphingolipid metabolism

1. Sphingomyelin
   - SM(d42:2)
   - SM(d44:1)
2. Galactosylceramide/Glucosylceramide (d40:1)

Ether lipid metabolism

1. Plasmenylcholine
   - PC(P-32:1)
   - PC(P-36:5)
   - PC(P-38:6)
   - PC(P-40:7)
2. 1-(1-Alkenyl)-sn-glycero-3-phosphocholine
   - LysoPC(P-16:0)
3. 1-(1-Alkenyl)-sn-glycero-3-phosphoethanolamine
   - LysoPEtn(P-16:0)
   - LysoPEtn(P-18:0)
4. O-1-Alk-1-enyl-2-acyl-sn-glycero-3-phosphoethanolamine
   - PEtn(P-36:5)
   - PEtn(P-38:5)
5. 1-Radyl-2-acyl-sn-glycero-3-phosphocholine
   - PC(O-36:1)

TABLE S1. Exemplified list of lipid species identified in human pleural effusion.

No.	Lipid classes	Lipid candidate	RT (min)	*m/z*	Adduct	ESI mode	Daughter m/z	Daughter m/z definition
**1.**	Fatty acids	FA(14:2)<br>5,8-Tetradecadienoic acid	0.78	223.170	[M-H]-	Neg	205.160<br>59.013	[M-H2O-H]-<br>[C2H3O2]-
		FA(16:2)<br>Hexadecadienoic acid	1.17	251.202	[M-H]-	Neg	59.013	[C2H3O2]-
		FA(18:1)<br>Oleic acid	2.65	281.249	[M-H]-	Neg	263.238	[M-H2O-H]-
		FA(18:2)<br>Linoleic acid	1.73	279.233	[M-H]-	Neg	261.223	[M-H2O-H]-
		FA(18:3)<br>Linolenic acid	1.25	277.217	[M-H]-	Neg	233.192<br>59.013	[M-CO2-H]-<br>[C2H3O2]-
		FA(20:3)<br>Eicosatrienoic acid	2.11	305.249	[M-H]-	Neg	261.259<br>59.013	[M-CO2-H]-<br>[C2H3O2]-
		FA(20:4)<br>Arachidonic acid	1.59	303.233	[M-H]-	Neg	259.243<br>285.223<br>59.013	[M-CO2-H]-<br>[M-H2O-H]-<br>[C2H3O2]-
		FA(20:5)<br>Eicosapentaenoic acid	1.16	301.217	[M-H]-	Neg	257.228<br>59.013	[M-CO2-H]-<br>[C2H3O2]-
		FA(22:4)<br>Adrenic acid	2.57	331.265	[M-H]-	Neg	287.275	[M-CO2-H]-
		FA(22:5)<br>Docosapentaenoic acid	1.79	329.248	[M-H]-	Neg	285.275<br>59.013	[M-CO2-H]-<br>[C2H3O2]-
		FA(22:6)<br>Docosahexaenoic acid	1.52	327.233	[M-H]-	Neg	283.243<br>59.013	[M-CO2-H]-<br>[C2H3O2]-
		FA(23:0)<br>Tricosanoic acid	6.02	353.343	[M-H]-	Neg	335.810	[M-CO2-H]-

No.	Lipid classes	Lipid candidate	RT (min)	*m/z*	Adduct	ESI mode	Daughter m/z	Daughter m/z definition
		HydroxylFA(16:0)<br>Hydroxy-hexadecanoic acid	0.89	271.228	[M-H]-	Neg	253.217<br>59.013	[M-CO2-H]-<br>[C2H3O2]-
		TrihydroxylFA(18:1)<br>Trihydroxy-octadecenoic acid	1.32	413.305	[M+2ACN+H]+	Pos	277.218	[M-2H2O+H]+
**2.**	Ceramides	GalCer(d40:1)/ GlcCer(d40:1)<br>Galactosyl-/Glucosyl-ceramide (d18:1/22:0)	8.09	842.674	[M+Hac-H]-	Neg	339.327<br>179.056<br>161.049	FA(22:0) [R2COO]-<br>[Hexone-H]-<br>[Hexone-H2O-H]-
		Gb3(d34:1)<br>Trihexosylceramide (d18:1/16:0)	6.17	1024.68	[M+H]+	Pos	538.520<br>520.510<br>502.499<br>264.269<br>282.279<br>682.563<br>844.615<br>1006.669<br>256.264	Loss of tri-hexones<br>Loss of tri-hexones and H2O<br>Loss of tri-hexones and 2H2O<br>[Sphingosine - 2H2O+H]+<br>[Sphingosine - H2O +H]+<br>Loss of di-hexone and H2O<br>Loss of hexone<br>[M-H2O+H]+<br>[FA(16:0)+NH3]+
		Gb3(d42:2)<br>Trihexosylceramide (d18:1/24:1)	7.82	1134.790	[M+H]+	Pos	264.269<br>282.279<br>648.630<br>630.618<br>612.608<br>792.672<br>972.735<br>954.725<br>1116.777<br>366.373	[Sphingosine - 2H2O+H]+<br>[Sphingosine - H2O +H]+<br>Loss of tri-hexones<br>Loss of tri-hexones and H2O<br>Loss of tri-hexones and 2H2O<br>Loss of di-hexone and H2O<br>Loss of hexone<br>Loss of hexone and H2O<br>[M-H2O+H]+<br>[FA(24:1)+NH3]+
		SM(d42:2)	8.01	1626.360	[2M+H]+	Pos	184.074<br>86.097<br>264.269<br>125.000	PC headgroup<br>Choline-H2O<br>[Sphingosine - 2H2O+H]+<br>Cyclophosphate

No.	Lipid classes	Lipid candidate	RT (min)	*m/z*	Adduct	ESI mode	Daughter m/z	Daughter m/z definition
		SM(d44:1)	8.41	841.7168	[M+H]+	Pos	184.074<br>86.097<br>125.000<br>264.269	PC headgroup<br>Choline-H2O<br>Cyclophosphate<br>[Sphingosine - 2H2O+H]+
**3.**	Lyso-Phospholipids	LysoPC(P-16:0)	1.34	480.345	[M+H]+	Pos	104.107<br>86.0970<br>184.074<br>125.000	Choline<br>Choline-H2O<br>PC headgroup<br>Cyclophosphate
		LysoPC(22:6)	0.82	568.34	[M+H]+	Pos	104.108<br>184.074<br>86.097<br>550.330<br>125.000	Choline<br>PC headgroup<br>Choline-H2O<br>[M-H2O+H]+<br>Cyclophosphate
		LysoPEtn(P-16:0)	1.50	436.283	[M-H]-	Neg	196.038<br>140.011<br>239.238<br>375.249	Glycerol PEtn-H2O<br>PEtn headgroup<br>P-16:0<br>[M-H-C2H7NO]-
		LysoPEtn(P-18:0)	3.49	464.315	[M-H]-	Neg	196.038<br>140.011<br>267.270<br>403.262<br>152.995	Glycerol PEtn-H2O<br>PEtn headgroup<br>P-18:0<br>[M-H-C2H7NO]-<br>1,2 cyclic phosphodiester
**4.**	Phosphatidyl Cholines	PC(34:2)<br>PC(16:0/18:2)	6.52	780.553	[M+Na]+	Pos	184.074<br>86.097<br>125.000<br>60.082<br>104.108<br>496.370<br>478.330<br>313.274	PC headgroup<br>Choline-H2O<br>Cyclophosphate<br>[NMe3]+<br>Choline<br>LysoPC(16:0)<br>LysoPC(16:0)-H2O<br>FA(16:0) [RCOO+58]+

**No.**	**Lipid classes**	**Lipid candidate**	**RT (min)**	***m/z***	**Adduct**	**ESI mode**	**Daughter m/z**	**Daughter m/z definition**
		PC(36:5)<br>PC(16:0/20:5)	6.17	802.536	[M+Na]+	Pos	597.488<br>86.097<br>184.073<br>313.273	[M-PC head]+<br>Choline-H2O<br>PC headgroup<br>FA(16:0)[RCOO+58]+
				838.560	[M+Hac-H]-	Neg	301.218<br>255.233<br>224.069	FA(20:5)[RCOO]-<br>FA(16:0)[RCOO]-<br>Phosphocholine (adducted)
		PC(41:6)	6.49	848.616	[M+H]+	Pos	184.074<br>86.097<br>104.108	PC headgroup<br>Choline-H2O<br>Choline
				906.622	[M+Hac-H]-	Neg	281.249<br>832.586	FA(18:1) [RCOO]-<br>[M-15]-
		PC(O-36:1)<br>PC(O-16:0/20:1)	8.00	832.665	[M+Hac-H]-	Neg	168.042<br>758.628<br>309.280<br>466.331	PC headgroup-CH3<br>[M-15]-<br>FA(20:1) [RCOO]-<br>LysoPC(O-16:0)
		PC(P-32:1)<br>PC(P-14:0/18:1)	6.34	716.559	[M+H]+	Pos	184.073<br>265.123<br>86.097	PC headgroup<br>FA(18:1) [RCO]+<br>Choline-H2O
				774.554	[M+Hac-H]-	Neg	168.042<br>281.249<br>419.838	PC headgroup-CH3<br>FA(18:1) [RCOO]-<br>[M-15]-[FA(18:1) R2COO]-
		PC(P-36:5)<br>PC(20:5/P-16:0)	6.43	764.559	[M+H]+	Pos	184.074<br>86.097<br>125.000<br>104.108	PC headgroup<br>Choline-H2O<br>Cyclophosphate<br>Choline
				822.539	[M+Hac-H]-	Neg	301.218	FA(20:5)[RCOO]-

No.	Lipid classes	Lipid candidate	RT (min)	*m/z*	Adduct	ESI mode	Daughter m/z	Daughter m/z definition
		PC(P-38:6)<br>PC(22:6/P-16:0)	6.67	790.575	[M+H]+	Pos	184.070<br>86.100<br>125.000	PC headgroup<br>Choline-H2O<br>Cyclophosphate
				848.582	[M+Hac-H]-	Neg	327.233<br>168.042<br>464.315	FA(22:6)[RCOO]-<br>PC headgroup-CH3<br>[LysoPC(P-16:0)-CH3]-
		PC(P-40:7)<br>PC(22:6/P-18:1)	6.74	816.589	[M+H]+	Pos	86.097<br>633.522<br>184.073<br>125.000	Choline-H2O<br>[M-PC headgroup]+<br>PC headgroup<br>Cyclophosphate
				874.596	[M+Hac-H]-	Neg	327.233<br>224.069<br>265.253<br>472.321	FA(22:6) [RCOO]-<br>Phosphocholine (adducted)<br>P-18:1<br>[LysoPC(P-18:1)-CH3]-
		PC(40:8)<br>PC(18:2/22:6)	6.06	830.568	[M+H]+	Pos	184.073<br>86.097<br>125.000<br>60.082<br>104.107	PC headgroup<br>Choline-H2O<br>Cyclophosphate<br>[NMe3]+<br>Choline
				888.576	[M+Hac-H]-	Neg	279.233<br>327.233<br>168.042<br>224.069	FA(18:2) [RCOO]-<br>FA(22:6) [RCOO]-<br>PC headgroup-CH3<br>Phosphocholine (adducted)
**5.**	Phosphatidyl Ethanolamines	PEtn(34:1)<br>PEtn(16:0/18:1)	7.19	716.524	[M-H]-	Neg	281.249<br>255.233<br>140.011<br>452.279	FA(18:1) [RCOO]-<br>FA(16:0) [RCOO]-<br>PEtn headgroup<br>LysoPEtn(16:0)

No.	Lipid classes	Lipid candidate	RT (min)	*m/z*	Adduct	ESI mode	Daughter m/z	Daughter m/z definition
		PEtn(36:4)<br>PEtn(16:0/20:4)	6.67	738.509	[M-H]-	Neg	303.233<br>255.233<br>140.011<br>452.279<br>196.038<br>259.243	FA(20:4) [RCOO]-<br>FA(16:0) [RCOO]-<br>PEtn headgroup<br>LysoPEtn(16:0)<br>Glycerol PEtn-H2O<br>[FA(20:4) -H-CO_2]-
		PEtn(38:4)<br>PEtn(18:0/20:4)	7.24	766.54	[M-H]-	Neg	303.233<br>283.265<br>259.243<br>140.011<br>196.038	FA(20:4) [RCOO]-<br>FA(18:0) [RCOO]-<br>[FA(20:4) -H-CO_2]-<br>PEtn headgroup<br>Glycerol PEtn-H2O
		PEtn(P-36:5)<br>PEtn(P-16:0/20:5)	6.56	720.497	[M-H]-	Neg	301.218<br>436.284<br>140.011	FA(20:5) [RCOO]-<br>LysoPEtn(P-16:0)<br>PEtn headgroup
		PEtn(P-38:5)<br><br>Mixture of<br>PEtn(P-18:0/20:5)<br>PEtn(P-16:0/22:6)	7.15	750.544	[M+H]+	Pos	609.525<br>359.258<br>392.290<br>387.290<br>364.262<br>294.316<br><br>266.285	Loss of PEtn headgoup<br>FA(20:5)[RCOO+58]+<br>[FA18:0 ether+C2H8NO3P]+<br>FA(22:5)[RCOO+58]+<br>[FA16:0 ether+C2H8NO3P]+<br>[FA18:0 ether+C2H8NO3P-H3PO4]+<br>[FA16:0 ether+C2H8NO3P-H3PO4]+
**6.**	Triacyl-glycerols	TG(53:7)	9.44	880.717<br>885.672<br>901.645	[M+NH4]+<br>[M+Na]+<br>[M+K]+	Pos<br>Pos<br>Pos	N/A	N/A
		TG(54:6)	9.79	900.793<br>915.742<br>931.761	[M+NH4]+<br>[M+Na]+<br>[M+K]+	Pos<br>Pos<br>Pos	N/A	N/A

No.	Lipid classes	Lipid candidate	RT (min)	*m/z*	Adduct	ESI mode	Daughter m/z	Daughter m/z definition
		TG(54:7)	9.53	894.755<br>899.711<br>915.684	[M+NH4]+<br>[M+Na]+<br>[M+K]+	Pos<br>Pos<br>Pos	N/A	N/A
		TG(54:8)	9.34	893.743<br>897.695<br>913.6687	[M+NH4]+<br>[M+Na]+<br>[M+K]+	Pos<br>Pos<br>Pos	N/A	N/A
		TG(56:8)	9.60	920.762<br>925.726<br>941.700	[M+NH4]+<br>[M+Na]+<br>[M+K]+	Pos<br>Pos<br>Pos	N/A	N/A
		TG(56:9)	9.37	918.755<br>923.713<br>939.684	[M+NH4]+<br>[M+Na]+<br>[M+K]+	Pos<br>Pos<br>Pos	N/A	N/A
		TG(58:10)	9.42	944.771<br>949.729<br>987.670	[M+NH4]+<br>[M+Na]+<br>[M+K]+	Pos<br>Pos<br>Pos	N/A	N/A
**7.**	Others	AcylCar18:2<br>Linoelaidyl carnitine	0.87	424.342	[M+H]+	Pos	85.0290	Acylcarnitine fragment ion $[C_4H_5O_2]^+$

FA, fatty acid; GalCer/GlcCer, galactosylceramide/glucosylceramide; Gb3, Trihexosylceramide; SM, sphingomyelin; LysoPC, lysophospahtidylcholine; LysoPC(P-), ether-linked lysophospahtidylcholine; LysoPEtn(P-), ether-linked lysophosphatidylethanolamine; PC, phosphatidylcholine; PEtn, phosphatidylethanolamine; PEtn(P-), ether-linked phosphatidylethanolamine; TG, triacylglycerols; Car, carnitine; M, molecular mass, Hac, acetic acid.
